# Performance of the FilmArray Blood culture identification panel in positive blood culture bottles and cerebrospinal fluid for the diagnosis of sepsis and meningitis

**DOI:** 10.3205/id000024

**Published:** 2016-09-09

**Authors:** Eva Leitner, Martin Hoenigl, Bernadette Wagner, Robert Krause, Gebhard Feierl, Andrea J. Grisold

**Affiliations:** 1 Institute of Hygiene, Microbiology and Environmental Medicine, Medical University of Graz, Austria; 2 Section of Infectious Diseases and Tropical Medicine, Department of Internal Medicine, Medical University of Graz, Austria

**Keywords:** FilmArray, blood culture, cerebrospinal fluid, multiplex PCR, sepsis, meningitis, rapid identification

## Abstract

Sepsis and meningitis are life threatening medical conditions. Culture-based methods are used for identification of the causative pathogens, but they can be improved by implementation of additional test systems. We evaluated the performance of the novel FilmArray blood culture identification (BCID; Biofire Diagnostics) panel for rapid and accurate identification of microorganisms in positive blood cultures and additionally, in this cerebrospinal fluid (CSF) pilot study for direct testing of CSF.

A total of 107 positive blood cultures and 20 CSF samples (positive and negative) were investigated and compared to the routine procedures.

Of the 107 positive blood cultures, 90.7% (97/107) showed monomicrobial growth and 9.3% (10/107) polymicrobial growth. The FilmArray BCID panel covered 89.3% (25/28) of the bacteria and 100% (2/2) of the yeasts found in this study and accurately identified all of them.

From the 20 retrospective analyzed CSF, in 9 positive specimens 6 different bacterial species were identified. Discrepant identification results were found in 25% (5/20) and a low sensitivity of 50% (95% CI of 15.7% to 84.3%) was detected.

Our study confirms the FilmArray BCID panel as a rapid, easy to handle PCR system with a good performance in positive blood cultures without Gram-staining result. However, our results additionally suggest that the system is not useful for direct CSF testing due to poor sensitivity.

## Introduction

Sepsis and meningitis are two serious life-threatening infections. The choice of empiric treatment in patients suffering from these conditions is calculated by means of actual guidelines based on underlying disease status and suspected infectious cause. Rapid diagnosis (i.e. rapid identification of the causative pathogen), followed by early modification of antimicrobial therapy (i.e. targeted therapy) is therefore essential to improve survival [1], [2], [3], [4].

In sepsis the leading bacteria causing this infection are Gram-positive cocci, especially *Staphylococcus aureus*, and Gram-negative bacilli, especially *Escherichia coli*. For bacterial diagnosis blood cultures (BC) represent still the gold standard for the detection and identification of pathogens [5], [6].

In meningitis the leading infectious agents are *Streptococcus pneumoniae*, *Neisseria meningitidis* and *Haemophilus influenzae*. For bacterial diagnosis of meningitis, direct Gram-staining and culture of cerebrospinal fluid (CSF) are mainly used and in addition latex agglutination tests can be employed for more rapid results [4].

For both diseases improvement of conventional culture-based identification systems is needed and new methods are available [7], [8], [9]. 

The recently introduced FilmArray blood culture identification (BCID) panel (BioFire, Salt Lake City, UT) an *in vitro* diagnostic (IVD)/Conformité Européene (CE)-labeled test system is a promising method for the use in positive blood cultures [10], [11]. It covers >90% of occurring pathogens in septic patients either to family, genus, or species level as well as the most important pathogens in bacterial meningitis [4], [5]. 

Previous studies have focused on evaluation of the test-system performance by using positive BC bottles incubated with blood or sterile body fluids. As an important limitation these studies did not find some microorganisms that are rarely isolated from blood cultures, which are covered by the BCID panel [12], [13], [14], [15]. These microorganisms rarely isolated from blood cultures included *L. monocytogenes*, *N. meningitidis* and *H. influenzae*, typical causative pathogens of bacterial meningitis that are more frequently cultivated from CSF [4]. As a sub aim we therefore decided to evaluate the performance of the BCID panel to detect these microorganisms in direct CSF specimens. Consequently, the aim of this study was to evaluate the FilmArray BCID identification panel in positive BC bottles and to evaluate for the first time in this CSF pilot study the usability for direct CSF testing.

## Materials and methods

### Ethical statement

Medical ethics review was not required for this study due to the fact that patients were not physically involved. In addition, privacy of patients was provided by coding all tested specimens. All specimens were taken as part of the standard hospital care and only residual specimens were included for testing.

### Sample collection 

One-hundred and ten positive blood culture bottles were obtained from the Institute of Hygiene, Microbiology and Environmental Medicine and the Department of Internal Medicine, Section of Infectious Diseases and Tropical Medicine, both Medical University of Graz, Austria. Samples were collected between November 2013 and July 2014. Blood cultures were obtained from patients with suspected sepsis as part of the standard hospital care and send to the respective laboratories. 

BACTEC Plus Aerobic/F or BACTEC Plus Anaerobic/F (Becton Dickinson Diagnostic Systems, Franklin Lakes, NJ, USA) blood culture bottles were included in this study when identified as positive by the automated blood culture-monitoring instrument BACTEC™ FX (Becton Dickinson). Only the first positive blood culture bottle that turned positive (from the three sets obtained routinely per patient) was included in this study. 

All CSF samples from patients with clinically suspected meningitis (community acquired or drainage associated) whether positive or negative in the routine procedure were frozen and analyzed retrospectively. The CSF was analyzed, if the residuary sample volume was sufficient for testing.

### Identification of microorganisms and resistance phenotypes

As reference method for both materials culture was used. Positive BCs were inoculated on agar plates according to the bottle type and Gram-staining result. Liquor specimens were centrifuged and pellet was inoculated on agar plates. From grown overnight cultures, identification was carried out depending on the suspected microorganisms either with VitekMS or Vitek2 (both; bioMérieux Marcy l’Etoile, France). When traditional methods failed, 16S rRNA-gene sequencing was used for identification. Susceptibility testing for resistance determination was done with disc diffusion and/or Vitek2 using EUCAST guidelines. All tests were performed at the Institute of Hygiene, Microbiology and Environmental Medicine, Medical University of Graz, which is an International Standard Organization (ISO 9001:2000) certified laboratory. 

### Bacteria and specimen preparation for FilmArray

For investigation from BC, 100 µl blood from a positive BC was transferred to the sample buffer.

To evaluate the limit of detection of main pathogens in meningitis for CSF testing, reference strains from American Type Culture Collection (ATCC) and a clinical isolate identified with VitekMS at the Institute of Hygiene, Microbiology and Environmental Medicine, Medical University of Graz were used: *Haemophilus influenzae* (ATCC 49247), Listeria monocytogenes (ATCC 19116), *Neisseria meningitides* (clinical isolate), and *Streptococcus pneumonia* (ATCC 49619). Following bacterial culture, stock suspensions of each bacterium were prepared in 0.85% NaCl with turbidity equivalent to a McFarland standard of 0.5 (corresponding to 1.5 x 10+E08 CFU/ml) and a 10-fold dilution series was prepared for testing until a concentration of 1.5 CFU/ml. From each suspension 500 µl were centrifuged at 3500 rpm for 20 minutes and a volume of 300 µl including pellet was transferred to the 500 µl sample buffer. Additionally to the FilmArray Gram-staining was performed. The latest positive results and the first negative result of the dilution series were tested in duplicate.

From residuary clinical CSF specimen’s containing a minimum of 500 µl, CSF was concentrated with centrifugation at 3500 rpm for 20 minutes. A volume of 300 µl CSF including the pellet was transferred with a pipette to the sample buffer.

### FilmArray processing 

The FilmArray BCID panel was used according to the manufacturer’s instructions. In brief, 100 µl of blood from the positive BC bottle or 300 µl of CSF (or diluted bacterial suspension) was transferred to the sample buffer. After mixing, the red-labeled syringe was filled with 300 µl of the mix and placed into the port of the pouch and pushed forcefully until hearing a “pop”. After verifying that the samples have been loaded the pouch were transferred to the FilmArray instrument and the run was initiated. When the run was finished the results were automatically displayed in a report. 

Two process controls are included in the assay. The DNA process control targets the yeast *Schizosaccharomyces pombe*, which is present in the pouch in a freeze-dried form and is introduced in the test when the sample is loaded. Therefore, it controls the whole FilmArray process from DNA extraction to PCRs. The positive result of this control indicates that all steps carried out in the pouch were successful. Additionally a PCR2 control is integrated, which detects a DNA target dried into the well of the array along with the corresponding primers and a positive result indicates that 2^nd^ stage PCR worked successfully. The run was regarded valid if the DNA process control and the PCR2 control targets were detected according to their ranges. For the comparison study only valid results were included.

## Results

### Analysis of positive blood cultures

After exclusion of 3 BC bottles (2 had invalid results, and in one Gram-stain, culture and FilmArray gave a negative result despite a positive signal in the blood culture system), a total number of 107 positive BC bottles were analyzed. 

With culture-based identification methods 28 bacterial and 2 fungal species were identified in 107 BCs. Of the 107 positive cultures 90.7% (97/107) showed monomicrobial growth and 9.3% (10/107) polymicrobial growth, respectively. In the 97 monomicrobial cultures 24 different microorganisms were detected and in the 10 polymicrobial cultures 13 different microorganisms were detected (Table 1 (Tab. 1)). As a consequence the FilmArray BCID panel covered 89.3% (25/28) bacteria and 100% (2/2) of the yeasts. Within the bacteria the FilmArray BCID panel identified 32.1% (9/28) to species level, 39.3% (11/28) to genus level and 17.9% (5/28) to family level, respectively. 105 of 107 positive BCs had microorganisms that were included in the FilmArray BCID panel, covering 97.6% (120/123) of clinical isolates during this study period either on species, genus or family level (Table 1 (Tab. 1)). 

The identified bacteria *Dolosigranulum pigrum* and *Bacillus* spp. were not covered from the FilmArray BCID panel. One *C. albicans* was only detected by the FilmArray BCID panel, but not by culture. Polymicrobial BCs were identified as polymicrobial in 90% (9/10) due to the composition of microbes, although not every bacterium was identified on species level. The polymicrobial growth of different coagulase-negative Staphylococci was not detected, although the identification result was correct (Table 2 (Tab. 2)). One error detected in a polymicrobial sample was that *Proteus vulgaris* was only identified correctly as member of Enterobacteriaceae, but was not identified as *Proteus* spp. In contrast, *Proteus mirabilis* in a monomicrobial sample was identified as *Proteus* spp.

Resistance genes were accurately detected in all samples (15/15). In 14 coagulase-negative Staphylococci the *mecA* was correctly identified and one *Enterococcus* spp. was positive for *vanA*/*vanB*, but no *bla*_KPC_-positive Enterobacteriaceae was detected in this study. 

### Analysis of CSF 

Detection limits were found to be 1.5 x 10E+05 CFU/ml for *Haemophilus influenzae* (ATCC 49247), 1.5 x 10E+04 CFU/ml for *Listeria monocytogenes* (ATCC 19116), 1.5 x 10E+04 CFU/ml for *Neisseria meningitides* (clinical isolate), and 1.5 x 10E+05 CFU/ml for *Streptococcus pneumonia* (ATCC 49619).

In the 20 clinical CFS specimens 6 different bacterial species were identified with culture-based methods: *L. monocytogenes* (n=2), *N. meningitides* (n=2), *S. epidermitis* (n=2), *S. haemolyticus* (n=1), *S. hominis* (n=1), and *S. pneumoniae* (n=1). The 20 clinical CSF specimens analyzed with both methods showed in 55% (n=11) a concordant negative result. In 20% (4/20) an accurate identification and in 25% (5/20) an inaccurate identification with the FilmArray BCID panel was observed. From the 5 inaccurate results, one was positive only with the FilmArray BCID panel and 4 were positive only in culture (Table 1 (Tab. 1)). 

Altogether, from the 20 CSF specimens, four were true positive, 11 true negative, one false positive and 4 false negative leading to a sensitivity of 50% (95% CI of 15.7% to 84.3%) and a specificity of 91.67% (95% CI of 61.5% to 99.79%) with a positive predictive value of 80% (95% CI of 28.36 % to 99.49%) and a negative predictive value of 73.3% (95% CI of 44.9% to 92.2%), respectively.

Altogether, in this study the FilmArray BCID panel was evaluated in specimens which included 89.5% (17/19) of bacteria, 40% (3/5) of yeasts and 66.7% (2/3) of resistance mechanisms covered by the system (“missing” pathogens or resistance mechanisms were *H. influenza*, *S. agalactiae*, *C. krusei*, *C. parapsilosis*, *C. tropicalis* and *bla*_KPC_-positive Enterobacteriaceae).

## Discussion

In our study, in BCs all 107 microorganisms included in the FilmArray BCID panel were accurately identified irrespective of mono- or polymicrobial growth. The major advantage of this system compared to other PCR-based methods is that a Gram-stain result is not mandatory to start with the test procedure due to the broad BCID panel spectrum, which additionally shortens the time to identification [12], [16]. In our study samples *E. coli* was the leading Gram-negative bacterium followed by *P. aeruginosa* and *K. pneumoniae* which are all covered by the FilmArray BCID panel on species level and were successfully identified in 100% of mono- and polymicrobial samples, comparable to other studies [14], [17]. Coagulase-negative staphylococci, *S. aureus* and *Enterococcus* spp. were the most frequently identified Gram-positive bacteria. All were identified correctly using the FilmArray, although it has to be mentioned that the polymicrobial growth of different coagulase-negative Staphylococci was not detectable. In general, detection of polymicrobial growth in the BC bottle with the FilmArray BCID panel depends on the composition of microorganisms and the ability to detect these species on species level. 

Another drawback of the FilmArray represents that *Enterococcus* spp. are identifiable to genus level only. Southern and colleagues have previously reported treatment strategies covering all Enterococci may help to overcome this drawback [11]. The two microorganisms not identified from the FilmArray BCID panel were *D. pigrum* and *Bacillus* spp., which are rarely present in septic patients. 

One discrepancy in our study was a false positive result of the FilmArray BCID panel compared to traditional culture, with the additional detection of *C. albicans* in a sample with monobacterial *S. haemolyticus* growth. In general, the fact that a PCR detects DNA regardless of the viable status of microorganisms has to be considered and clinical interpretation may be challenging in some cases. Nevertheless, the inclusion of yeasts in one test panel together with bacteria is very beneficial due to known difficulties in the diagnosis of candidemia, which is a concern worldwide [11], [18], [19], [20]. 

Furthermore, resistance genes were accurately detected in all 15 samples. In this study 14 *mec*A genes were detected in coagulase-negative Staphylococci only. The false detection of *mec*A shown by Bhatti and colleges was not seen [12]. The detection of a vancomycin resistant *E. faecium* based on the ability of the FilmArray BCID panel to detect *vanA/vanB* during this study underlines the usefulness of integrated resistance genes which can have an important impact for treatment adaption in septic patients [11]. 

The detection limits shown for the mentioned microorganisms around 10E+05 CFU/ml should be sufficient using this method for CSF from positive BC bottles, but is critical when used for direct CSF testing [21]. A recent publication describes the first use of the FilmArray BCID panel for direct testing of 19 CSF specimens presenting a sensitivity of 73% and a specificity of 100% [22]. In our small pilot study (20 specimens) we detected a sensitivity of 50% and specificity of 92%, respectively, which is lower than in the study by Mico et al. The low concordance between the FilmArray BCID panel and routine procedure might be from the result of the high detection limit and the fact, that only residual specimens were available for this retrospective analysis and prior treatment for routine procedures might have decreased bacterial load in these CSF specimens. 

Recently a new panel specifically for meningitis diagnosis was introduced, the FilmArray Meningitis-Encephalitis (ME) Panel that covers the most important viruses and bacteria in meningitis as well as *Cryptococcus* spp. requiring 200 µl CSF volume. Evaluation studies of the new panel did not retest the analytical sensitivity. The clinical specimens showed a low detection rate of bacteria and yeasts including discrepant results with the used reference method [23], [24], [25]. Hanson KE concludes that the new panel characteristics are acceptable for clinical care, but pathogen detections should be scrutinized carefully and cannot replace Gram stain and culture [26].

One limitation of this study was the low number of samples positive for some microorganisms in blood and in direct CSF samples. Additionally, no specimen was positive for *H. influenzae*, *S. agalactiae*, *C. krusei*, *C. parapsilosis*, *C. tropicalis* and *bla*_KPC_-positive Enterobacteriaceae and performance of the FilmArray BCID panel for these bacterial species could therefore not be evaluated. Another limitation is the fact that using the FilmArray BCID panel exclusively in positive BCs needs to consider the limitations of the BC itself like slow growth rate and a high negative rate of the cases where true bacterial or fungal sepsis is believed to exist [27]. 

Furthermore, quality control is an issue for most multiplex PCRs on the market, including the FilmArray system [10], [12], [28]. So far, for molecular diagnostics the inclusion of a positive, negative and an internal control per PCR run was mandatory [29], [30]. The control offered by the manufacturer of FilmArray is the process control replacing the internal control only. The challenge is that for multiplex PCRs multiplex positive controls as well as multiplex external quality controls should be applied in defined intervals but there are no providers or guidelines available so far. 

As described in other studies the rapid time to identification (within 1h) and the easy handling are crucial advantages of the system; however, the high costs and the possibility to process only one sample at a time on the same instrument needs a careful consideration before implementing this method [13], [14]. We are in line with Altun et al. that it could be a useful tool in hospitals with a BC instrument without a microbiological laboratory to improve the time to result in positive flagged BCs due to the easy work-up [14]. The use of the FilmArray BCID panel is limited as suggested to the use in BCs only due to the insufficient sensitivity for direct CFS specimens. 

To conclude, the FilmArray BCID panel showed a convincing performance as a rapid, easy to handle PCR system for the use in positive blood cultures without knowledge of the Gram-stain result. In contrast, our results suggest that the system is not useful for direct CSF testing due to poor sensitivity. 

## Notes

### Conference presentations

Data of the manuscript have been previously presented, in part, at 24^th^ European Congress of Clinical Microbiology and Infectious Diseases (ECCMID) 2014 (poster presentation 0530), and at the 10^th^ International Symposium on Molecular Diagnostics (ISMD) 2014 (poster presentation 07).

### Competing interests

The authors declare that they have no competing interests.

### Acknowledgements

We thank BioFire for providing equipment and price rebates for the FilmArray assays in this study. 

## Figures and Tables

**Table 1:**
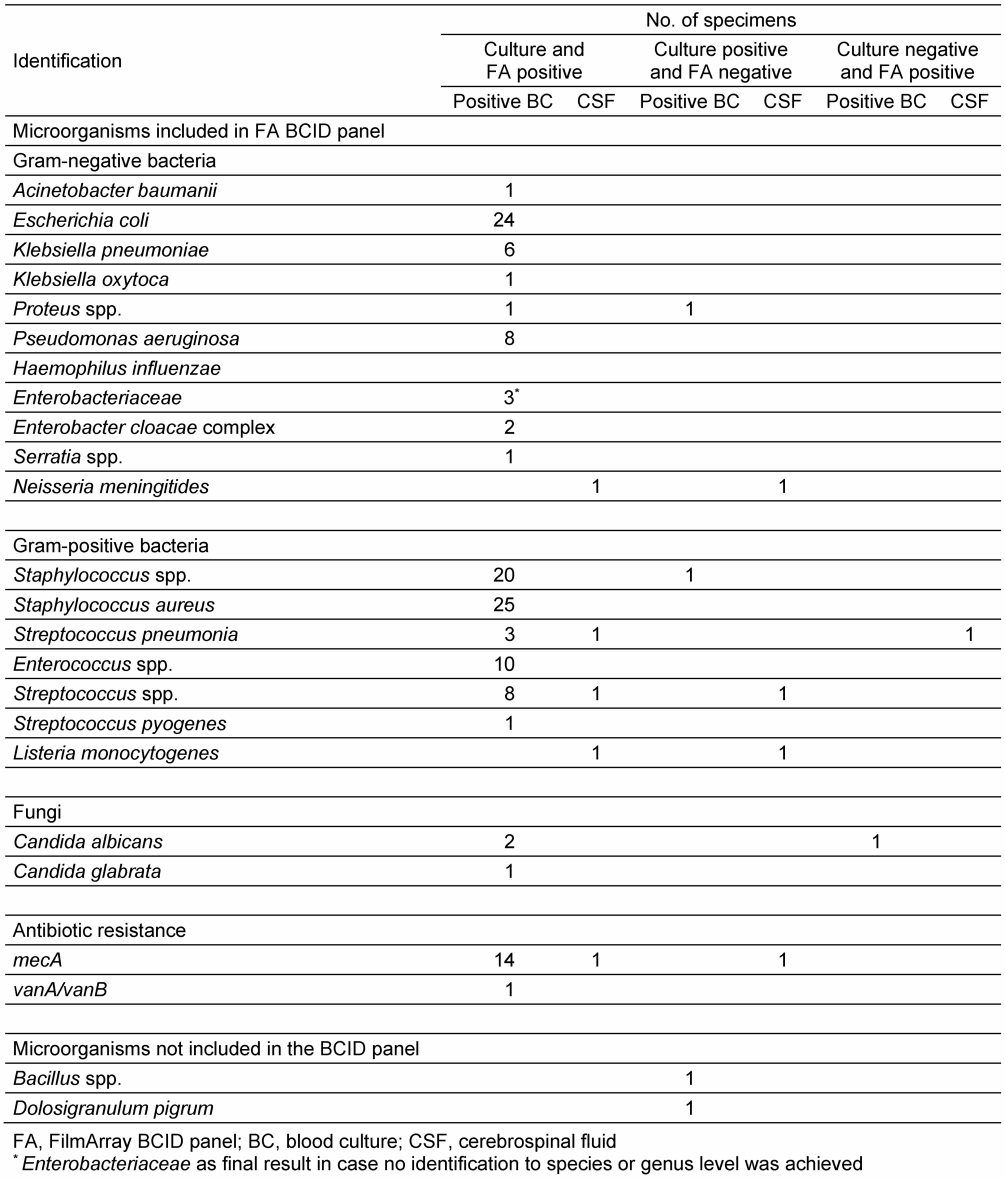
Identification of pathogens and antibiotic resistance marker with culture and FilmArray BCID panel from positive blood cultures and cerebrospinal fluid samples

**Table 2:**
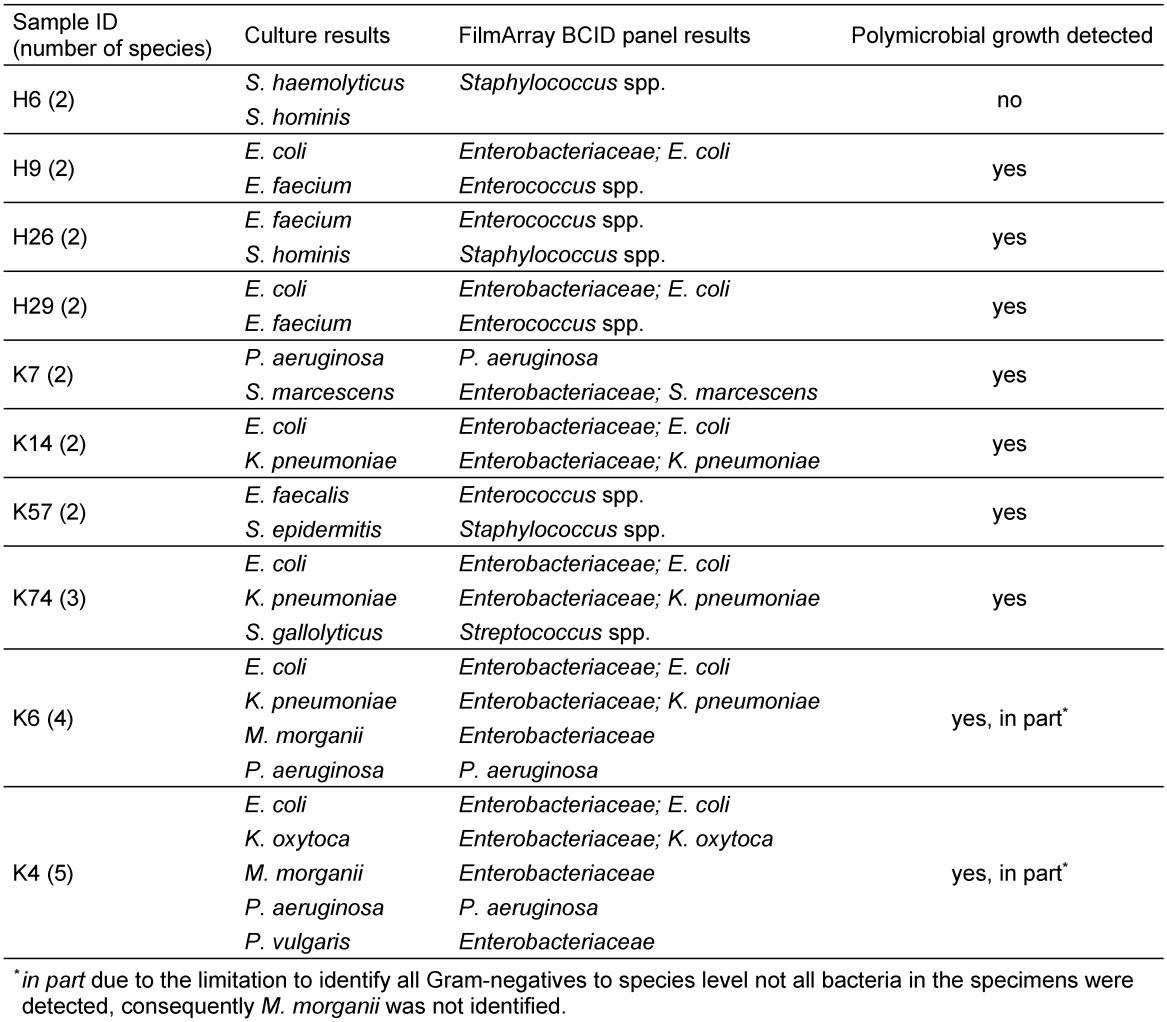
Performance in identification of polymicrobial blood cultures with the FilmArray BCID panel
